# Sea ice and millennial-scale climate variability in the Nordic seas 90 kyr ago to present

**DOI:** 10.1038/ncomms12247

**Published:** 2016-07-26

**Authors:** Ulrike Hoff, Tine L. Rasmussen, Ruediger Stein, Mohamed M. Ezat, Kirsten Fahl

**Affiliations:** 1 CAGE—Centre for Arctic Gas Hydrate, Environment and Climate, Department of Geology, UiT, The Arctic University of Norway, NO-9037 Tromsø, Norway; 2 Alfred Wegener Institute, Helmholtz Centre for Polar and Marine Research, D-27568 Bremerhaven, Germany; 3 Department of Geosciences (FB5), Klagenfurter Strasse 4, University of Bremen, 28359 Bremen, Germany; 4 Department of Geology, Faculty of Science, Beni-Suef University, Beni-Suef, Egypt

## Abstract

In the light of rapidly diminishing sea ice cover in the Arctic during the present atmospheric warming, it is imperative to study the distribution of sea ice in the past in relation to rapid climate change. Here we focus on glacial millennial-scale climatic events (Dansgaard/Oeschger events) using the sea ice proxy IP_25_ in combination with phytoplankton proxy data and quantification of diatom species in a record from the southeast Norwegian Sea. We demonstrate that expansion and retreat of sea ice varies consistently in pace with the rapid climate changes 90 kyr ago to present. Sea ice retreats abruptly at the start of warm interstadials, but spreads rapidly during cooling phases of the interstadials and becomes near perennial and perennial during cold stadials and Heinrich events, respectively. Low-salinity surface water and the sea ice edge spreads to the Greenland–Scotland Ridge, and during the largest Heinrich events, probably far into the Atlantic Ocean.

## Abstract (web summary)

The response of Arctic sea-ice to rapid climatic change in the past remains uncertain. Here, the authors use biomarkers and microfossils to reconstruct Arctic sea-ice changes over the past 90,000 years, and demonstrate millennial-scale variability.

## Graphical abstract

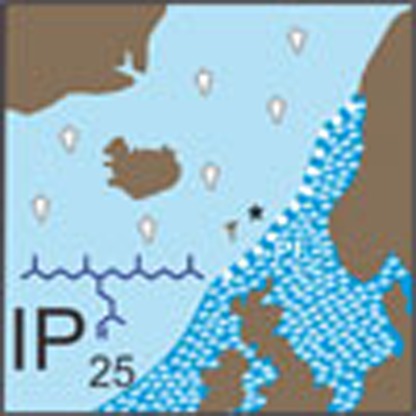

Dansgaard/Oeschger (D/O) events in Greenland ice cores consist of warm interstadial (IS) and cold stadial events^1^ and are strongly imprinted in sediments from the northern North Atlantic region and Nordic seas^2^,^3^. In general the warming to insterstadial conditions was abrupt as seen in Greenland ice cores and marine records. The warm conditions were followed by gradual cooling called the insterstadial transitional cooling phase, and a rapid transition to cold stadial conditions. Larger and/or longer-lasting stadials correlate with North Atlantic Heinrich events (H-events)^2^, where numerous icebergs were released from the Laurentide ice sheet and melting over the North Atlantic region in the so-called Ruddiman belt^4^,^5^ (Fig. 1). Even though D/O events have been extensively studied, changes in sea ice cover have only been inferred by indirect evidence for presence or absence of sea ice (for example, deposition patterns of ice-rafted debris, oxygen isotope records and palaeo-temperature reconstructions) (Supplementary Fig. 1 and Supplementary Table 1).

The Nordic seas are characterized by northward inflow of warm, saline Atlantic surface Water (North Atlantic Current, Northwest Atlantic Current, North Atlantic surface Water, Faroe Current) and southward outflow of cold Polar surface Water (East Greenland Current and East Icelandic Current)^6^ (Fig. 1). In the Fram Strait, the Atlantic Water continues its flow below the sea ice-covered Polar surface Water as an intermediate water mass^7^. In the central part of the Nordic seas cooling and sinking of the salty surface water during the winter months generate cold deep overflows over the Greenland–Scotland Ridge into the North Atlantic^6^,^7^. The inflow of Atlantic surface Water is the major source of heat to the Arctic and Nordic seas, and it is generally agreed that changes in ocean circulation and sea ice cover has played a major role in the control of past millennial-scale climate changes of the glacial D/O events^2^,^8^,^9^.

The Atlantic surface Water is ice-free throughout the year, while the East Greenland Current is covered by drifting near-perennial sea ice. In the central parts of the Nordic seas, mixing of Atlantic Water with Polar Water forms the zone of Arctic surface Water, which is located between the Arctic and Polar fronts^6^ (Fig. 1). The Arctic surface Water is seasonally sea ice covered and comprises the marginal ice zone (MIZ). The location of the MIZ and the Arctic and the Polar fronts changes with the seasons and on inter-annual and longer-time scales^10^. In the East Greenland Current behind the Polar front productivity is very low, while intermediate to high productivity is found in the ice-free zone of Atlantic surface Water. The highest seasonal productivity occurs at the frontal areas and in the MIZ^11^,^12^. The positions of the Arctic and Polar fronts and the degree of sea ice cover thus depend on the distribution of the major surface water masses in the Nordic seas. A recent study showed that in the Arctic Ocean, the flow of Atlantic Water has a direct impact on sea ice distribution^13^.

Previous studies of a C_25_ isoprenoid lipid (IP_25_) synthesized mainly by diatoms have shown its potential as a valuable new proxy for the reconstruction of the presence of seasonal sea ice^14^,^15^,^16^,^17^,^18^,^19^. IP_25_ reportedly is produced by a few sea ice diatom species within the genera *Haslea* and *Pleurosigma*^20^. Furthermore, IP_25_ is a stable organic compound preserved in sediments as old as Late Miocene and Pliocene^21^,^22^,^23^, and can be used in environments where other micropalaeontological sea ice proxies are absent or disturbed by dissolution effects^14^,^24^. Open-ocean conditions have been successfully reconstructed by using phytoplankton-derived sterols as a proxy for increased surface productivity such as brassicasterol and dinosterol^15^,^25^,^26^. The calculated ratio IP_25_ to brassicasterol and dinosterol, the so-called P_B_IP_25_ and P_D_IP_25_, respectively, can be related to sea ice coverage on a scale from permanent sea ice to year-round open water. In both ends of the scale, the IP_25_ proxy is practically absent^15^,^16^,^26^, but accompanied by either low or high brassicasterol and dinosterol values, respectively^26^,^27^.

In this study we present results of IP_25_, brassicasterol, dinosterol, total organic carbon (%TOC) and δ^13^C_org_ (as terrigenous/marine organic-matter proxy; see ref. 28) together with the calculated sea ice indicators P_B_IP_25_ and P_D_IP_25_, in the interval 801–2 cm of sediment core JM11-FI-19PC from the SE Norwegian Sea (see Methods and Fig. 1 for core location). In addition, low-resolution counts and identification of diatom frustules have been performed (see Methods). The purpose of this study is to reconstruct sea ice cover in the past in relation to millennial-scale climate change during the last 90 kyr in medium to high resolution. We compare our records with previously published records from the Fram Strait and published records that have provided indirect evidence for sea ice distribution and ocean circulation on millennial timescale from the northern North Atlantic (Fig. 1, Supplementary Fig. 1 and Supplementary Table 1). The chosen core site is from the northern Faroe Islands margin in the SE Norwegian Sea, close to the position of previously published core ENAM93-21/MD95-2009 (ref. 3). The site is close to the Iceland–Faroe Front (IFF) that marks the boundary between the cold East Icelandic Current branching off the East Greenland Current and the Faroe Current branch of the inflowing Atlantic surface Water^6^ (Fig. 1). The northern Faroe margin is presently sea ice free year round, but historical records show that during the Little Ice Age sea ice drift reached to the Faroe Islands via the East Icelandic Current^29^.

Our study shows that the sea ice retreats abruptly at the start of warm Interstadials and spreads rapidly during cooling phases of the Interstadials, before it becomes near-perennial and perennial during cold stadials and H-events, respectively. The distribution of sea ice correlates closely with climate and variations in ocean circulation, in flow of meltwater and stratification of the ocean surface.

## Results

### Correlation and age model

The age model for the last 90 kyr interval of sediment core JM11-FI-19PC is based on well-known tephra layers (Figs 2 and 3 and Supplementary Table 2), 11 AMS-^14^C dates (Fig. 3 and Supplementary Table 3) and 20 magnetic susceptibility (MS), potassium/titanium (K/Ti) and δ^18^O tie points of insterstadial onsets (Fig. 3 and Supplementary Table 4). It has previously been demonstrated that the signal of MS and benthic δ^18^O values in marine records from the SE Nordic seas correlate closely in time with the (North) Greenland ice core δ^18^O signal^3^,^30^ (Fig. 2). The characteristic saw-tooth pattern of the D/O events in the ice cores is recognized in the MS of our core (Fig. 2). High values correlate with insterstadial and minimum values with stadial (S) climate, respectively. The presented interval of JM11-FI-19PC comprises the interstadials IS21–IS1 (IS1=Bølling and Allerød interstadials^1^) and H-events H8–H1 (refs 8, 31), which is equivalent to the last 90 kyr b2k (before year AD 2000) (Figs 2 and 3).

The age control of the core JM11-FI-19PC younger than 65 kyr b2k has been presented by Ezat *et al*.^30^, while the age model for the interval older than 65 kyr b2k is new (see Methods and Supplementary Information for details). Sample resolution in the studied interval 90 kyr ago to present (per 1-cm thick sample) varies between 35 and 320 years, while the sedimentation rates vary between 0.28 mm per year (Fig. 3).

### IP_25_ and other biomarkers

**Figure 1:**
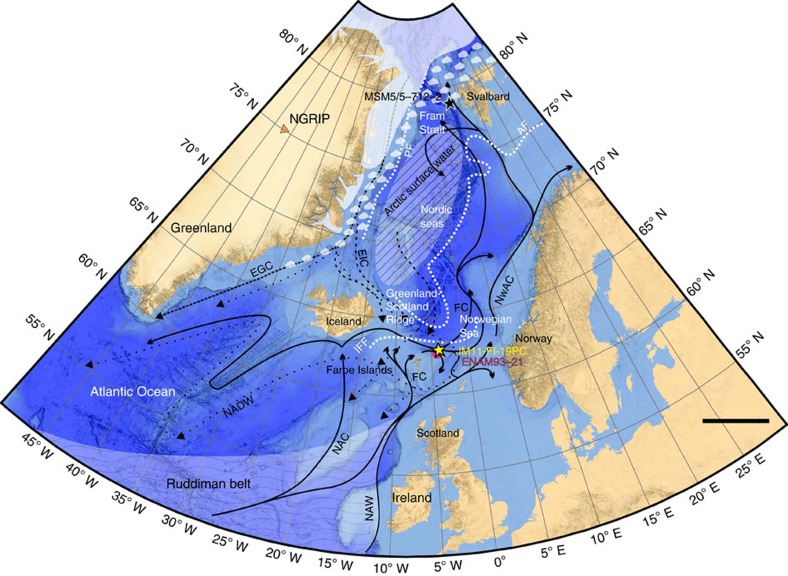
Map of the Nordic seas. Location of studied sediment core JM11-FI-19PC (yellow star) along with nearby core ENAM93-21/MD95-2009 (refs 3, 66, 67, 68) (magenta coloured circle) and core MSM5/5-712-2^17^ (black star) from the Svalbard margin, discussed in the text, are marked. Bathymetry from GEBCO 2014 grid (http://www.gebco.net/). Major surface (solid and dashed black lines) and bottom currents (dotted black lines), locations of Arctic Front (AF) including the Iceland-Faroe front (IFF)) and Polar Front (PF) (dashed white lines) are indicated together with the modern location of summer sea ice limit (shaded white area with drift ice), and the location of the Arctic surface water (shaded area with diagonal lines), as well as the Ruddiman belt (shaded area with wave-shaped lines). EGC, East Greenland Current; EIC, East Icelandic Current; FC, Faroe Current; NAC, North Atlantic Current; NADW, North Atlantic Deep Water; NAW, North Atlantic Water; NwAC, Northwest Atlantic Current; NGRIP, North Grip ice core (orange triangle). Scale bar, 500 km.

For the last 30,000 years, trends in our PIP_25_ record are similar to core MSM5/5-712-2 from the western Svalbard margin (for core location see Fig. 1) with very similar IP_25_, P_B_IP_25_ and P_D_IP_25_ values^17^ (Fig. 4). In the Svalbard record, maxima in sea ice cover occurred in marine isotope stage (MIS) 2 (between 30 and 17 kyr ago), while the sea ice cover was slightly reduced after 17 kyr ago in the warm Bølling and Allerød interstadials (IS1) and in the Holocene^17^ (Fig. 4). A very similar development is seen at the northern Faroe margin and with the same events clearly marked. The similarity of the data indicates that the sea ice proxy results for the Faroe margin are overall reliable. The minor offsets in the timing of the IP_25_, to P_B_IP_25_ and P_D_IP_25_ might reflect uncertainties in the age models and the fact, that the Svalbard record is located in the direct flow of Atlantic Water, while JM11-FI-19PC is also influenced by the IFF (Fig. 1).

For the part older than 30 kyr, sea ice variability (reflected in the IP_25_, P_B_IP_25_ and P_D_IP_25_ records) and related short-term changes in surface-water productivity (reflected in the brassicasterol and dinosterol records and in the flux patterns of diatoms) seem to follow very clearly the D/O events (Fig. 5).

## Discussion

In late MIS 3 to early MIS 2 (c. 35–22 kyr ago), the interstadials of D/O events 6–2 are short-lasting, and because of the relatively low sampling resolution in JM-FI-19PC in this time interval (>300 years per sample) not all the interstadials are equally well resolved (Fig. 5; see also ref. 30). In the following discussion we therefore focus on the time interval 90–35 kyr ago comprising the longer-lasting D/O events 21–7 with the highest resolution, including H-events H8–H4 (Fig. 5). A total of three different phases within each D/O event could be determined; insterstadial conditions, insterstadial transitional cooling conditions and stadial conditions (Fig. 5). A fourth phase describes the particular conditions during some larger H-events (H6, part of H4 and H1; Fig. 5).

Interstadials are characterized by absence or near absence of IP_25_, maximum content of brassicasterol and dinosterol, and low P_B_IP_25_ and P_D_IP_25_ values (Fig. 5). The (near) absence of IP_25_ indicates absence of sea ice-related diatoms, while high brassicasterol and dinosterol values indicate favourable conditions for phytoplankton growth in general and higher primary productivity at the surface ocean. *Thalassiosira oestrupii* (Ostenfeld) Hasle, an indicator of relatively warm temperatures^32^,^33^ is present in higher amounts in IS21 (MIS 5a) and in the Holocene, coinciding with high values of brassicasterol and dinosterol (Fig. 5, see Methods for details on diatoms). The species occurs sporadic or is absent in interstadials of MIS 3 and 2 (IS17–IS3) indicating colder than modern conditions. Altogether, the biomarker signals indicate open-ocean conditions and relatively high surface water temperatures over the northern Faroe Islands margin, with the Arctic Front located north of the core position (Fig. 6a). Planktic foraminiferal species from nearby core ENAM93-21/MD95-2009 indicated inflow of Atlantic Water during interstadials of MIS 3 and MIS 2^3^ in support of the interpretation based on diatom floras and organic biomarkers. Furthermore, the δ^13^C_org_ values are higher in interstadials, which point to a (relative) decrease of terrigeneous organic matter supply and/or an increase in marine organic matter input^28^. The latter option, that is, an increased influence of marine organic matter at times of higher water temperatures due to higher marine productivity at the core location, is supported by higher flux of diatoms and higher concentrations of brassicasterol and dinosterol (Figs 5 and 6a).

The optimum conditions are followed by a cooling phase defined by gradual changes to medium or higher values of IP_25_, brassicasterol, and dinosterol, P_B_IP_25_ and P_D_IP_25_ (Fig. 5). The relatively higher P_B_IP_25_ and P_D_IP_25_ values together with the increase in brassicasterol and dinosterol values (most clearly seen in IS21, IS20, IS13–10 and IS8; marked by arrows in Fig. 5) indicate increasing seasonal sea ice cover and elevated surface productivity, respectively, typical of the marginal ice zone^11^,^12^ (Fig. 6b). These findings suggest that the IFF had moved to a probably variable position south-east of the core location and that the area was in the zone of Arctic surface water, likely resulting in decrease of both atmospheric and surface-water temperatures (Fig. 6b). This phase correlates with an increase in ice rafting and an increase in cold-water planktic foraminiferal species as seen in nearby core ENAM93-21/MD95-2009 (ref. 3).

The cooling phase terminated in a phase of extended sea ice cover. This is associated with a maximum in IP_25_ values (higher than the calculated mean value), an abrupt decrease in the concentration of brassicasterol, dinosterol and high P_B_IP_25_ and P_D_IP_25_ values (Fig. 5, light-blue colour bars). Generally supporting this, the sea ice-associated *Fragilariopsis oceanica* (Cleve) Hasle^33^,^34^,^35^ is present in higher relative abundance in H-event 8, S16 and S1 (Fig. 5, see Methods for details on diatoms). The lack of diatoms in most of the identified phases of extended sea ice cover (Fig. 5) is in accordance with the low abundances of diatoms found in areas with near-perennial sea ice cover today^16^,^20^ (for diatom preservation, see Methods). With cold surface conditions and extended sea ice cover as far south as the core location, the Polar Front migrated to a position close to or probably south of the core site (Fig. 6c). Conditions were probably similar to conditions currently observed in areas governed by the East Greenland Current with (very) low primary productivity^36^,^37^ and a dense pack-ice cover. Rather low δ^13^C_org_ values might indicate decreased marine organic carbon flux due to the low productivity and/or an increased influence of terrigenous organic matter, probably being ice-rafted to the core site. The presence of near-perennial sea ice prevented heat exchange and the atmosphere was probably very cold. These last phases of the D/O events represent the stadial conditions of the Greenland ice cores.

The sea ice proxy data for the H-events 6, 4 and 1 (Figs 4 and 5) show a very prominent period of perennial or near-perennial sea ice cover (absent or medium IP_25_, respectively, absent or minimal brassicasterol and dinosterol, and maximum P_B_IP_25_ and P_D_IP_25_ values). In these intervals, the IP_25_ values of zero cannot be interpreted as absence of sea ice. Instead, the combined signals likely represent a thick permanent sea ice cover and very cold temperatures^15^,^26^ (see also discussion in ref. 17). As long as the sea ice cover is thin enough for sunlight to penetrate, sea ice diatoms synthesizing IP_25_ can grow attached beneath the ice^14^,^16^,^20^, which was probably the case at the beginning and end of some of the stadial intervals, where high peaks in P_B_IP_25_ and P_D_IP_25_ can occur (Fig. 5). When the sea ice becomes permanent and too thick for sunlight to penetrate, the signal of IP_25_ will drop to zero (Fig. 5). To allow for the perennial sea ice and extreme cold temperatures, Polar surface water most likely spread over the area north of the Faroe Islands and the Polar Front was located far more southerly than today and during the smaller stadials (Fig. 6d).

The transition from stadial and H-events to interstadials is rapid and interpreted as a sudden transition from extended sea ice cover to open-ocean conditions, seen as a decrease in values of IP_25_, P_B_IP_25_ and P_D_IP_25_ (with the exception of H6, H4 and H1; Fig. 5). The abrupt decrease in sea ice cover is in line with the abrupt increase in atmospheric temperatures at the beginning of D/O events^1^ and increase in sea surface temperatures as also seen in nearby record ENAM93-21/MD95-2009 (ref. 3).

Our data generally show a good correlation between climate and sea ice cover for MIS 3, as well as for the last 30,000 years (Figs 4 and 5). We demonstrate that the presence/absence of sea ice varies closely in pace with the different climatic phases of the D/O millennial-scale climate events (Fig. 5). The peak warm interstadials with no sea ice (Fig. 7a) (Supplementary Fig. 1 and Supplementary Table 1) resembled the modern conditions, which have been shown by numerous marine core studies from the northern North Atlantic and Nordic seas, and have generally been interpreted as a sign of strong flow of Atlantic surface water (Fig. 6a). The transitional cooling phase of the insterstadial with gradually expanding sea ice cover from northwest (Figs 6b and 7b) correlate with increase in ice rafting from icebergs, decreasing atmospheric temperatures^1^ and an increasing amount of meltwater over a larger area of the northern North Atlantic region and Nordic seas. In the following stadial events iceberg rafting reached a maximum (Figs 6c and 7c) as also seen in nearby core ENAM93-21/MD95-2009 and other records from the Nordic seas and North Atlantic (Supplementary Fig. 1 and Supplementary Table 1), and δ^18^O values reached very low values indicating presence of meltwater (Fig. 5). Sea ice advanced and the sea ice cover became near-perennial in the case of smaller stadial events (Figs 6c and 7c) or perennial as during the colder H-events H6, H4 and H1 (Figs 6d and 7d). The latter events are the three strongest events during the last 90 kyr, probably due to orbital forcing^2^,^38^. All stadial and H-events in the North Atlantic and Nordic seas show dominance by the polar planktic foraminifera *Neogloboquadrina pachyderma* sinistral (s) and cold polar conditions (see references in Supplementary Information; Supplementary Fig. 1 and Supplementary Table 1).

Sea ice was an active player in millennial climate change, in most cases probably enforcing trends already caused by the predominantly cold, glacial atmospheric conditions during the last glacial period. The peak warmth of the interstadials lasted only shortly^1^ and was immediately followed by cooling and spreading of sea ice. The inflow of Atlantic surface Water to the core area diminished to the extent that deep-water formation became very slow and stopped and sea ice cover became perennial or near perennial. The ocean circulation in the Nordic seas was probably more similar to the system in the northern Fram Strait today, in our view the closest analogue to the situation during stadials and North Atlantic H-events^38^ and with similar circulation patterns of water masses with warmer Atlantic water flowing at intermediate depth below Polar surface Water^38^,^39^,^40^,^41^. In other words, the present-day conditions of the Fram Strait moved far south into the Atlantic Ocean (Fig. 7c,d). The remarkable abrupt disappearance of sea ice at the end of stadials/H-events correlates with sudden renewed inflow of Atlantic surface Water, peak insterstadial warmth and probably onset of deep-water formation. The distribution of sea ice thus correlated closely with variations in ocean circulation and expanding-retreating ice sheets and variations in flow of meltwater and stratification of the ocean surface.

## Methods

### Core logging and sampling

The core handling, sampling and diatom flora analyses of sediment core JM11-FI-19PC (62°49.97 N; 03°52.03 W; 1179, m water depth; 1109, cm core recovery) were performed at the Department of Geology, UiT, Arctic University of Norway, Tromsø, Norway. Before opening the magnetic susceptibility (MS) was measured with a Bartington MS meter MS2 (loop; sample resolution: 1 cm). After opening of the core, but before sampling, it was scanned for light and heavy elements, such as potassium (K) or titanium (Ti) with an Avaatech XRF core scanner in a resolution of 0.5–1 cm (ref. 30).

Samples taken at 5-cm intervals in 1-cm thick slices were weighed, freeze-dried and weighed again before wet sieving over 63 and 100-μm sieves. Tephra particles were counted in the size fraction >100 μm. The method for counting tephra particles followed the procedures described in Wastegård and Rasmussen^42^.

Subsamples at 5-cm intervals from the presented core section (801–2 cm) were used to determine TOC. For %TOC dried and powdered aliquots of the samples were treated with 10% hydrochloric acid (HCl), for carbonate removal, before being measured with a Leco CS-200.

### Carbon isotopes of organic material

Carbon isotopes δ^13^C_org_ were measured every 5 cm in a powdered and carbonate-free aliquot of the samples, using a Finnigan MAT Delta-S mass spectrometer equipped with a FLASH elemental analyser and a CONFLO III gas mixing system for the online determination of the carbon isotopic composition. Measurements were conducted at the Alfred Wegener Institute Helmholz Centre for Polar and Marine Research in Potsdam, Germany. The s.d. (1*σ*) is generally better than δ^13^C=±0.15‰.

### Oxygen isotopes in planktic foraminifera

Oxygen isotopes were measured on specimens of the planktic foraminiferal species *N. pachyderma* s. For this, about 30 specimens in the size fraction 150–250 μm were analysed with a Finnigan MAT 251 mass spectrometer with an automated carbonate preparation device. Measurements were performed at the Department of Geosciences, at the University of Bremen, Germany. The external standard error of the oxygen isotope analyses is δ^18^O=±0.07‰. Values are reported relative to the Vienna Pee Dee Belemnite, and calibrated by using the National Bureau of Standards NBS18, 19 and 20.

### Quantification of diatom floras

The preparation method for diatom samples given in Koç *et al*.^43^ was followed, but using hydrogen peroxide (H_2_O_2_ 37%; 10 drops) instead of nitric acid (HNO_3_ 65%) to remove existing organic matter around the diatom valves. Quantitative diatom slides were produced as described in Koç Karpuz and Schrader^44^. A sample was considered to contain enough diatoms for quantification, when there were more than 40 diatom valves (=20 frustules) within ∼350 fields of view (2 vertical transects) and a minimum of 300 diatom valves per sample were counted. A total of 52 species that previously have been used by Koç Karpuz and Schrader^44^ for establishing a sea-surface-temperature-transfer function (including additional species added by Andersen *et al*.^45^) have been identified and counted on a *Chaetoceros* spp. free basis^44^,^46^. Diatom counts followed the procedures of Schrader and Gersonde^47^ and taxonomic identification mainly followed those of Hustedt^48^,^49^, Fryxell and Hasle^50^,^51^, Simonsen^52^, Hasle and Fryxell^53^, Hasle^54^, Syvertsen^55^, Sancetta^56^ and Sundström^57^.

For the interval between 78 and ∼15 kyr b2k numerous fragmented frustules were found. This might indicate that this interval was influenced by dissolution processes^58^, which additionally could have altered the species composition of the diatom flora, and/or, that the sediments were directly influenced by the presence of sea ice as a mechanical component, breaking diatom valves by shear forces^59^. Diatom data for this interval have thus to be considered with caution and our reconstruction of past sea ice cover is exclusively based on the biomarker proxies that are more resistant and preserved in this type of sediments^14^,^16^,^23^ (for general aspects of preservation of biomarkers see refs 28, 60). Between ∼15 kyr b2k and the present the preservation of diatom frustules is excellent.

### Biomarker analyses

The lipid biomarker analyses were performed at the Alfred Wegener Institute Helmholtz Centre for Polar and Marine Research, Bremerhaven, Germany. Freeze-dried and homogenized sediments (4–5 g) were extracted with an Accelerated Solvent Extractor (DIONEX, ASE 200; 100 °C, 5 min, 1000, psi) using a dichloromethane:methanol mixture (2:1 v/v). Before this step, 7-hexylnonadecane, squalane and cholesterol-d_6_ (cholest-5-en-3β-ol-d_6_) were added as internal standards. Hydrocarbons and sterols were separated via open column chromatography (SiO_2_) using *n*-hexane (5 ml) and methyl-acetate:*n*-hexane (20:80 v/v, 6 ml), respectively. Sterols were silylated with 500 μl BSTFA (60 °C, 2 h)^61^. For qualification and quantification of IP_25_ and sterols see Fahl and Stein^62^. The detection limit for IP_25_ is 0.01 ng on column. P_B_IP_25_ and P_D_IP_25_ were calculated from IP_25,_ brassicasterol and dinosterol concentrations, respectively, according to Müller *et al*.^26^.

It is important, when using the PIP_25_ index to distinguish between different sea ice conditions, that coevally high amounts of both biomarkers (suggesting ice-edge conditions) as well as coevally low contents (suggesting permanent-like ice conditions) would give a similar or even the same PIP_25_ value. Especially, for the latter situation of permanent sea ice conditions both biomarker concentrations may approach values around zero and the PIP_25_ index may become indeterminable (or misleading). For a correct interpretation of the PIP_25_ data the individual IP_25_ and phytoplankton biomarker concentrations must be considered^15^,^26^. Recently, Smik *et al*.^63^ introduced a HBI-III alkene as phytoplankton biomarker replacing the sterols in the PIP_25_ calculation. This modified PIP_25_ approach is based on biomarkers from the same group of compounds (that is, HBIs) with more similar diagenetic sensitivity, which is important for palaeo-sea ice reconstructions and comparison of records from different Arctic areas.

### Radiocarbon dating

Eleven AMS-^14^C dates of piston core JM11-FI-19PC have previously been presented by Ezat *et al*.^30^. They were performed on monospecific samples of the planktic foraminiferal species *N. pachyderma* s (Supplementary Table 3). Measurements were performed at the ^14^CHRONO Centre for Climate, the Environment, and Chronology, at Queens University Belfast, Northern Ireland. For this study all ^14^C ages were calibrated to calendar years using the CALIB Radiocarbon Calibration 7.0.2. software and the Marine13 data set (including 400 year correction for surface reservoir ages)^64^,^65^. To make the age scale comparable to the ice core timescale (GICC05) 50 years were added. All ages are in ice core years b2k (before year AD 2000).

### Construction of the age model

**Figure 2:**
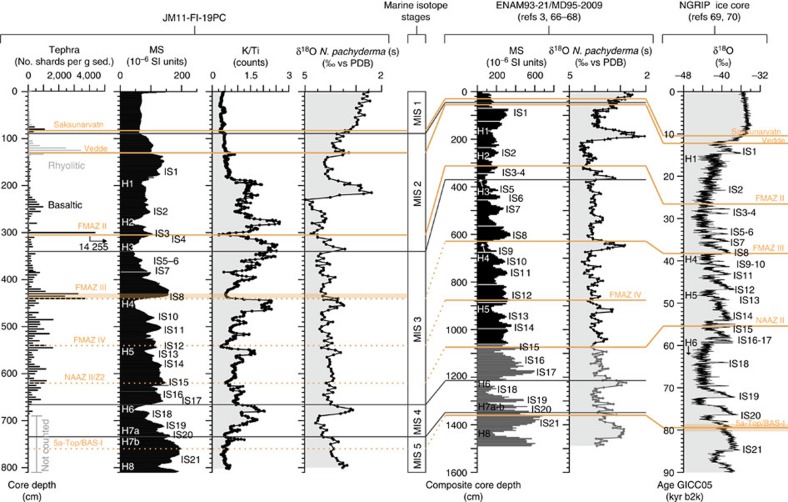
Stratigraphic correlation of JM11-FI-19PC to nearby marine core ENAM93-21/MD95-2009 and the NGRIP ice core. Tephra layers are shown in orange (solid lines, used in age model calculations; dashed lines, not used in age model calculations; see explanations to Supplementary Table 2). Basaltic tephra counts for JM11-FI-19PC are shown in black bars, while rhyolitic tephra counts are shown in light grey bars. Orange shaded bar at the FMAZ III tephra from core JM11-FI-19PC indicate depth range of the tephra zone (see explanations to Supplementary Table 2). Orange shaded bar at the 5a-Top/BAS-I tephra layer in data from the NGRIP ice core indicates uncertainty in age for this tephra layer (see explanations to Supplementary Table 2). H, H-event; K/Ti, XRF measurement of the ratio between potassium and titanium; MS, Magnetic susceptibility; δ^18^O *N. pachyderma* s, planktic foraminiferal species. Data from sediment (sed.) cores ENAM93-21/MD95-2009 are from refs 3, 66, 67, 68, while NGRIP ice core data are from refs 69, 70.

**Figure 3:**
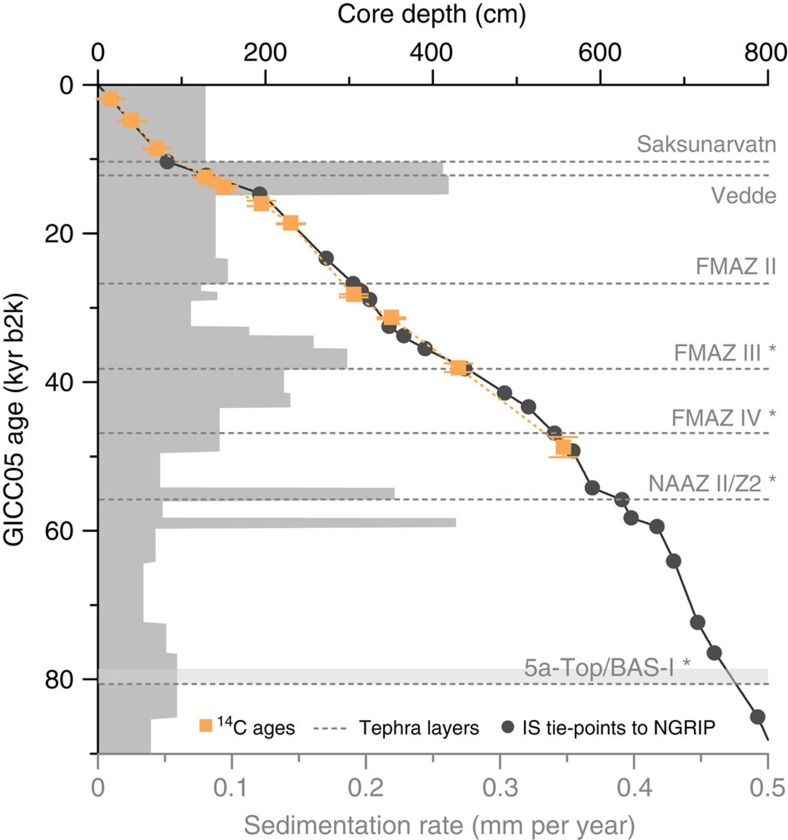
Age–depth relationship and sedimentation rates of the upper 801 cm of sediment core JM11-FI-19PC. Tephra layers marked with * (FMAZ III, IV, NAAZ II/Z2 and 5a-Top/BAS-I) have not been used in the calculations of the age model (see also Methods and explanations to Supplementary Table 2). Error bars are given in s.d.

**Figure 4:**
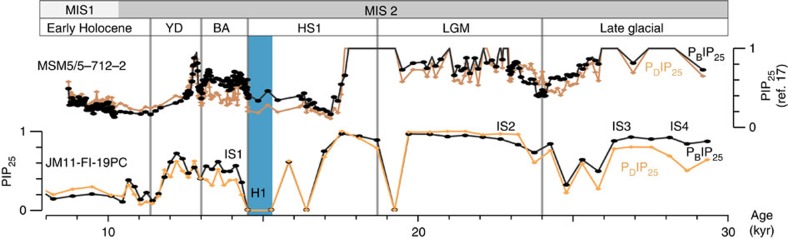
Sea ice proxies for the time interval between 30 and 8 kyr ago. Comparison of P_B_IP_25_ and P_D_IP_25_ signals of sediment core JM11-FI-19PC (GICC05-timescale) and sediment core MSM5/5-712-2 (calibrated age scale) from the Svalbard margin published by Müller and Stein^17^. For H-event 1 (blue bar), characterized by IP_25_ and brassicasterol (as well as dinosterol) values of close to or zero (that is, ‘zero divided by zero'), per definition maximum P_B_IP_25_ and P_D_IP_25_ values of 1 were assumed (for background see refs 15, 26). For core locations, see Fig. 1. BA, Bølling and Allerød interstadial; H, H-event; HS, Heinrich stadial; LGM, Last Glacial maximum; YD, Younger Dryas.

**Figure 5:**
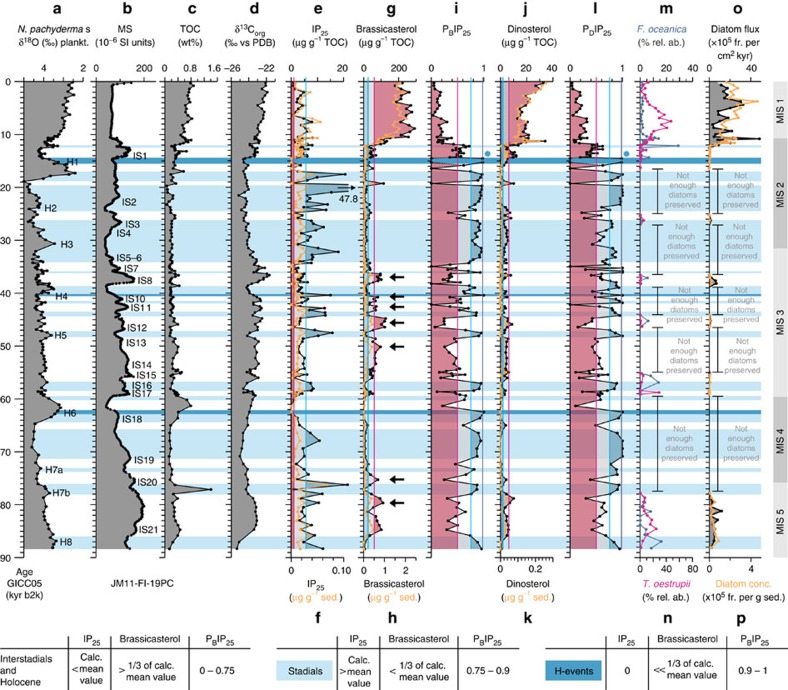
Proxy data of sediment core JM11-FI-19PC 90 kyr b2k to present. (**a**) δ^18^O measured in planktic foraminiferal species *N. pachyderma* s (‰ versus (vs) Pee Dee Belemnite (PDB)), (**b**) MS (10^−6^ SI units), (**c**) TOC (% of dry weight sediment), (**d**) δ^13^C_org_ (‰ vs PDB), (**e**) IP_25_ (μg g^−1^ TOC, black) and (**f**) (μg g^−1^ sediment (sed.), orange), (**g**) brassicasterol (μg g^−1^ TOC, black) and (**h**) (μg g^−1^ (**i**) calculated index P_B_IP_25_ (brassicasterol), (**j**) dinosterol (μg g^−1^ black) and (**k**) (μg g^−1^, (**l**) calculated index P_D_IP_25_ (dinosterol), (**m**) relative abundance of diatom species *Fragilariopsis oceanica* (Cleve) Hasle (%, blue), (**n**) relative abundance of diatom species *Thalassiosira oestrupii* (Ostenfeld) Hasle (%, magenta), (**o**) diatom flux (10^05^ frustules (fr.) per cm^2^ per kyr, black) and (**p**) diatom concentrations (10^05^ frustules per g sed., orange), and MIS are shown. Vertical lines in IP_25_, brassicasterol and P_B_IP_25_ mark the cutoff values of sea ice cover stages^26^,^27^. Legend at the bottom of the figure show cutoff values of IP_25_ (μg g^−1^ TOC), brassicasterol (μg g^−1^ TOC) and P_B_IP_25_ as criteria of the interpretation of IP_25_, brassicasterol, dinosterol, P_B_IP_25_ and P_D_IP_25_ in terms of degree of sea ice cover. Brassicasterol values (as proxy for open-water phytoplankton productivity) are in great accordance with the dinosterol numbers, therefore only values for brassicasterol are shown. Zero or near-zero concentrations of both IP_25_ as well as phytoplankton biomarkers are indicative for a closed (spring) sea ice cover (PIP_25_ in general is indeterminable and per definition set to ‘1' (refs 15, 26); see P_B_IP_25_ and P_D_IP_25_ during H1, marked by blue stars). Arrows mark events of increased productivity during IS cooling phases (see text for explanation). H, H-event; ‘not enough diatoms preserved', in these intervals there were not enough diatoms preserved to be quantified after the set criteria (see Methods).

**Figure 6:**
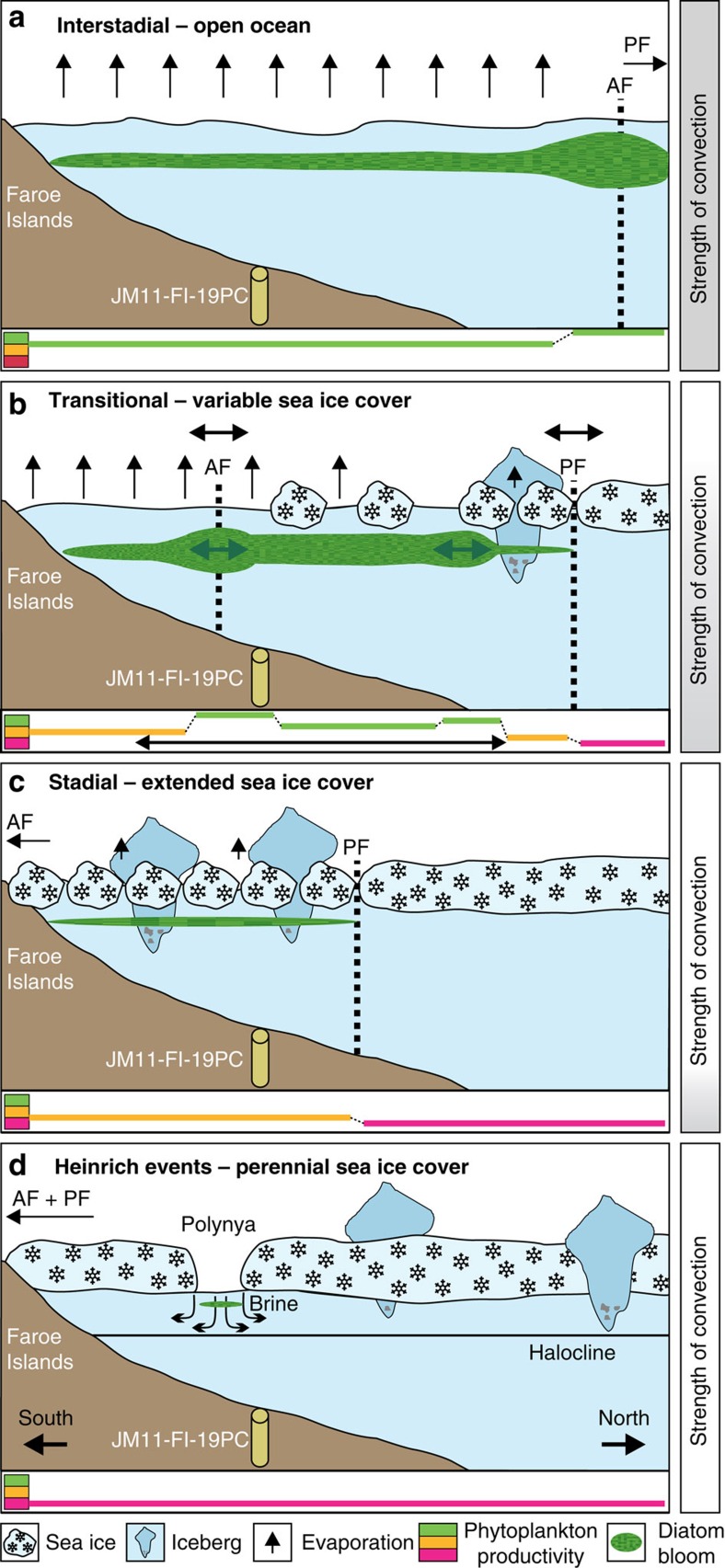
Schematic reconstruction of sea ice conditions at the northern Faroe slope. (**a**) Interstadial. (**b**) Interstadial transitional cooling phase. (**c**) Stadial. (**d**) H-events H6, H4 and H1. Black horizontal arrows indicate location of the Arctic Front (AF) and Polar Front (PF). Green colour (including arrows) indicates degree of phytoplankton productivity inferred from concentrations of brassicasterol, dinosterol and flux of diatoms. Productivity levels are indicated in the lowermost part of each figure: Magenta coloured line, no or very little phytoplankton productivity; yellow line, medium/variable productivity; green line, high or very high productivity. Grey vertical bars on the right indicate strength of deep convection.

**Figure 7:**
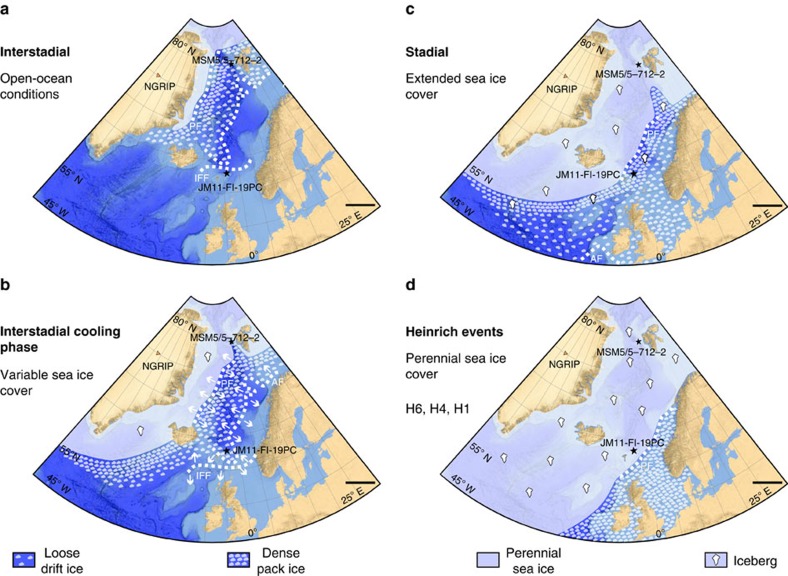
Reconstructed sea ice distribution during D/O events in the Nordic seas. The figure is based on published records with sufficient time resolution to identify individual D/O events of MIS 5 to MIS 2. We have included only records that contain counts of ice-rafted debris (IRD) in combination with planktic δ^18^O values and/or surface temperature proxies based on planktic foraminifera (transfer functions and/or % *N. pachyderma*). From areas that are well covered with a high number of records, we have selected a few considered as representative. For areas with sparse coverage and low resolution (such as central basins), we chose a few records, where the results may be based on other or fewer proxies (Supplementary Table 1). For locations of the published records and references, see Supplementary Fig. 1. (**a**) Interstadial with open-ocean conditions. The Arctic Front (AF), Iceland-Faroe Front (IFF) and Polar Front (PF) are located north of the core location. (**b**) Interstadial transitional cooling phase with variable sea ice cover and variable positions of AF (IFF) and PF, which is indicated by white arrows. The core location is always between AF and PF. (**c**) Stadial with extended sea ice cover. The core location is again between AF and PF, but markedly closer to PF (potentially PF can be south of the core location; see text). Both fronts are located further towards the south compared with interstadial and interstadial cooling conditions. (**d**) H-events (H6, H4 and H1) with perennial sea ice cover. Both Fronts, AF and PF, are now located south of the core location. Stars mark the location of the studied core JM11-FI-19PC, as well as the location of core MSM5/5-712-2^17^ used for comparison; dashed white lines give approximate positions of fronts AF (IFF) and PF. The reconstructions of regional distribution of sea ice cover are based on published records together with the records of JM11-FI-19PC and MSM5/5-712-2^17^ (Supplementary Fig. 1, Supplementary Table 1 and Supplementary References). Bathymetry from GEBCO 2014 grid (http://www.gebco.net/). NGRIP, North Grip ice core (orange triangle). Scale bar, 500 km.

The age model younger than 65 kyr is based on the age model published in Ezat *et al*.^30^. The age model from 90 to 65 kyr b2k was constructed by using the same approach and at the same time validating the age model of the younger sediments published by Ezat *et al*.^30^ (Supplementary Tables 2, 3 and 4). To further solidify the age model, piston core JM11-FI-19PC was not only correlated to the NGRIP ice core but also to the nearby compiled marine cores ENAM93-21 (ref. 3) and MD95-2009 (refs 66, 67) based on the MS, common tephra layers and stable oxygen isotopes (δ^18^O) measured in the planktic foraminiferal species *N. pachyderma* s (Fig. 2; see also Supplementary Table 2). The ages in between the tie points and/or tephra layers were calculated by interpolation assuming a constant sedimentation rate (Fig. 3).

### Data availability

Data referenced in this study are available in PANGAEA with the identifier DOI ‘ https://doi.org/10.1594/PANGAEA.859992'.

## Additional information

**How to cite this article:** Hoff, U. *et al*. Sea ice and millennial-scale climate variability in the Nordic seas 90 kyr ago to present. *Nat. Commun.* 7:12247 doi: 10.1038/ncomms12247 (2016).

## Supplementary Material

Supplementary Information Supplementary Figure 1, Supplementary Tables 1-4 and Supplementary References.
