# Supplementary material for: Sea ice and millennial-scale climate variability in the Nordic seas 90 kyr ago to present
Source: Nat Commun. 2016 Jul 26;7:12247. doi: 10.1038/ncomms12247 (PMC4963477; doi:10.1038/ncomms12247)
Supplement: Supplementary Information — Supplementary Figure 1, Supplementary Tables 1-4 and Supplementary References. [file ncomms12247-s1.pdf]

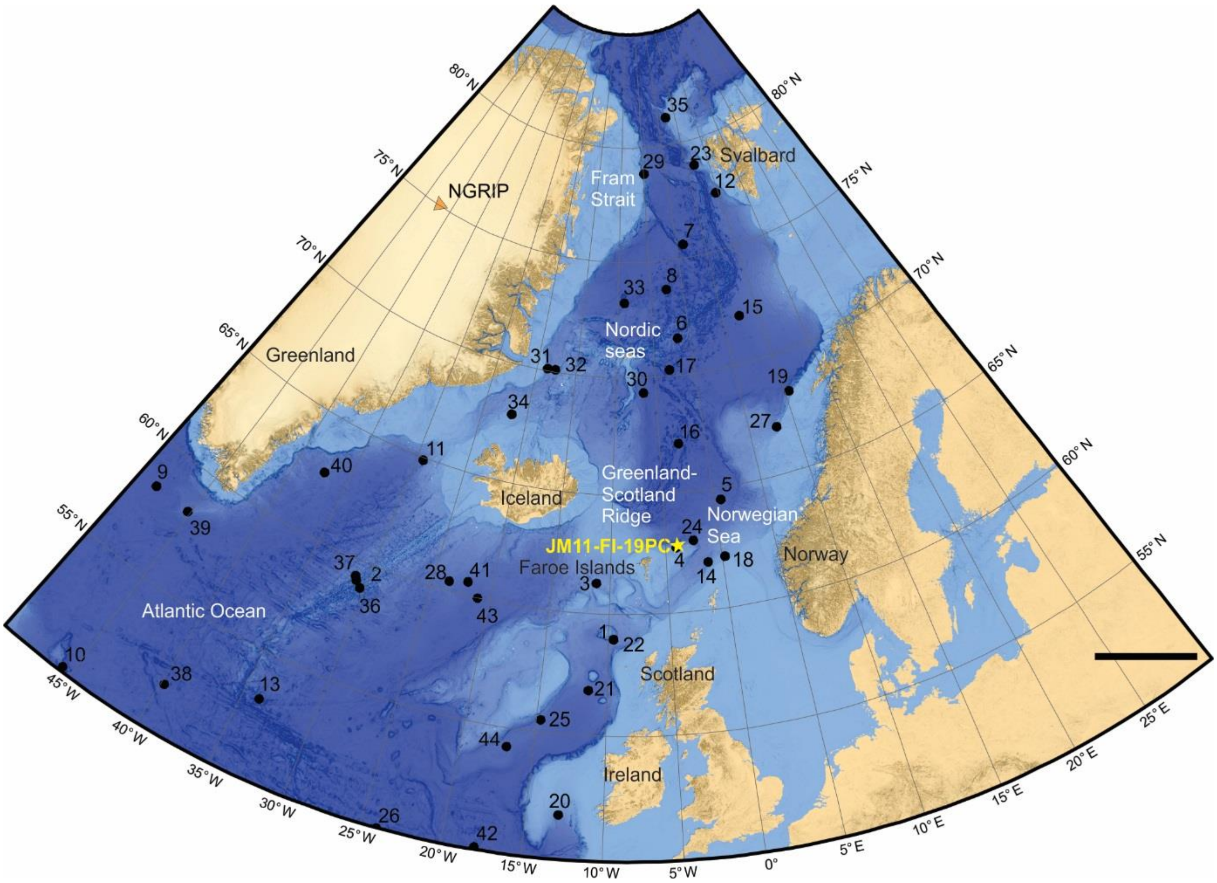

Supplementary Figure 1. Locations of cores used in reconstruction of sea ice in the northern North Atlantic and Nordic seas. The numbers refer to the numbers listed in Supplementary Table 1. A yellow asterisk marks the investigated core JM11-FI-19PC. The location of the North Greenland ice core (NGRIP) is indicated by an orange triangle. Bathymetry from GEBCO 2014 grid (http://www.gebco.net/). Scale bar represents 500 km.

Supplementary Table 1. Core names, positions and references for published records shown in Supplementary Fig. 1 and used for the reconstruction of sea ice shown in Fig. 7a-d.

No	Core name	Proxy for temperature	Reference
1	DAPC-02	% *N. pachyderma* s IRD, planktic isotopes	1
2	DS97-2P	% *N. pachyderma* s, $\delta^{18}O$, foraminiferal SST, IRD	2, 3
3	ENAM33	% *N. pachyderma* s, IRD, planktic isotopes	4
4	ENAM93-21	% *N. pachyderma* s, IRD, planktic $\delta^{18}O$	5
5	HM52-43	Planktic $\delta^{18}O$, diatom SST	6
6	HM94-13	Planktic $\delta^{18}O$, diatom SST	6
7	HM94-25	Planktic $\delta^{18}O$, SST	7
8	HM94-34	Planktic $\delta^{18}O$, SST	7
9	HU-90-013-013	Planktic $\delta^{18}O$, dinoflagellate SST, and sea ice	8, 9
10	HU-91-045-094	Planktic $\delta^{18}O$, dinoflagellate SST, and sea ice	8, 9
11	JM96-1225	% *N. pachyderma* s, planktic $\delta^{18}O$, IRD	10
12	JM04-025PC	$\delta^{18}O$, IRD	11
13	JPC-13	Planktic $\delta^{18}O$, IRD	12
14	LINK17	% *N. pachyderma* s, planktic $\delta^{18}O$	13
15	M17730	Planktic $\delta^{18}O$, foraminiferal SST	7
16	M23071	Planktic $\delta^{18}O$, foraminiferal SST	7
17	M23259	Planktic $\delta^{18}O$, foraminiferal SST	7
18	MD99-2284	Planktic $\delta^{18}O$, IRD, foraminiferal SST	14
19	MD99-2294	% *N. pachyderma* s, planktic $\delta^{18}O$, IRD, foraminiferal SST	15
20	MD01-2461	% *N. pachyderma* s, planktic $\delta^{18}O$, IRD, foraminiferal SST	16
21	MD04-2822	% *N. pachyderma* s, IRD	17
22	MD04-2829CQ	% *N. pachyderma* s, planktic $\delta^{18}O$, foraminiferal SST	18
23	MSM5/5-712-2	IP_{25}, P_BIP_{25} and P_DIP_{25}	19
24	NA81-10	% *N. pachyderma* s, planktic $\delta^{18}O$, IRD	20
25	NA87-22	Planktic $\delta^{18}O$, IRD, foraminiferal SST	21
26	ODP Site 609	% *N. pachyderma* s, planktic $\delta^{18}O$	22
27	ODP Site 644	% *N. pachyderma* s, planktic $\delta^{18}O$, IRD	23
28	ODP Site 983	% *N. pachyderma* s, IRD	24
29	PS1230	% *N. pachyderma* s, planktic $\delta^{18}O$, IRD	25
30	PS1243	% *N. pachyderma* s, planktic $\delta^{18}O$, IRD	25
31	PS1726	Planktic $\delta^{18}O$, IRD	26
32	PS1730	Planktic $\delta^{18}O$, IRD	27, 28
33	PS1878	% *N. pachyderma* s, planktic $\delta^{18}O$, IRD	29
34	PS2644	% *N. pachyderma* s, planktic $\delta^{18}O$, IRD	30
35	PS2837-5	IP_{25}, PIP_{25}	31
36	SO82-02GGC	Planktic $\delta^{18}O$, IRD, foraminiferal SST	32
37	SO82-05GGC	% *N. pachyderma s*, planktic $\delta^{18}O$, IRD, foraminiferal SST	33, 34
38	SU90-12	Isotopes, IRD, foraminiferal SST	21
39	SU90-16	Isotopes, IRD, foraminiferal SST	21
40	SU90-24	Foraminiferal SST, IRD	35
41	SU90-33	Planktic $\delta^{18}O$, IRD, foraminifera SST	21
42	SU90-44	Planktic $\delta^{18}O$, IRD, foraminifera SST	21
43	V29-202	% *N. pachyderma* s, planktic $\delta^{18}O$, IRD	36
44	VM23-81	% *N. pachyderma* s	22
	NGRIP		37, 38
	JM11-FI-19PC	IP_{25}, P_BIP_{25} and P_DIP_{25}, diatoms	This study

Supplementary Table 2: Tephra layers, GICC05 ages and depth of occurrence in NGRIP ice core and marine core JM11-FI-19PC. * FMAZ-III was excluded from the age model as Griggs *et al*. (ref. 39) found, that in marine records this layer is a mixture of the tephra from numerous volcanic eruptions. ** FMAZ-IV is located in the lower part of IS12[40]. It has not yet been located in the NGRIP ice core and has therefore not been used in the age model; although Griggs *et al*. (ref. 39) found, that it can be used as isochron for high-precision correlations. *** Ash Zone II (Z2) has not been used in the age model, as the peak in counted shards has not been considered distinctive enough[40]. **** Due to its wide range in age, the 5a5a-Top/BAS-I tephra layer has not been included in the final age model; although identified in all cores used in this study. Nevertheless, all listed tephra layers have been used for formal correlation (Fig. 2). Depth marked with ▲ were determined/approved by counting micro-tephra shards whilst identifying/counting diatom species.

Reference horizon	GICC05 age ± 1σ (yr b2k)	NGRIP depth (m)	Reference (NGRIP)	JM11-FI-19PC depth (cm)	Reference (JM-11-FI-19PC)
**Saksunarvatn tephra**	10 347 ± 45	1409.83	37	83	41
**Vedde tephra /NAAZ I**	12 171 ± 57	1506.14	37	130	41
**FMAZ II / Fugloyarbanki tephra**	26 740 ± 390	1848.05	42	305	41
**FMAZ III tephra ***	38 122 ± 723	2066.95	38	427-428<br>438-439	39
**FMAZ IV tephra ****	46 800 ± 1000	-	40	542-543	39, 41
**NAAZ II/ Z2 tephra *****	55 380 ± 1184	2359.45	38	620▲	41, This study
**5a-Top/BAS-I *******	78 500 - 80 100	2600.60	43, 44, 45	760▲	This study

Supplementary Table 3: AMS-^{14}C dates. Conventional and calibrated radiocarbon dates of core JM11-FI-19PC. Ages were first converted to calendar years before present (1950) using the CALIB Radiocarbon Calibration 7.0.2. Software and the Marine13 data set (including 400 year correction for surface reservoir ages)[46, 47], before 50 years were added to make them comparable to the ice core time scale GICC05[38] in b2k (before 2000 years). ▲ Marks the depth used for establishing the age model by Ezat *et al*. (ref. 41) and in this study. All other AMS-^{14}C dates are used to control the reliability of the age model only (Fig. 3).

**Depth (cm)**	**Material**	**Laboratory Code**	**Age (^{14}C a yr BP)**	**Age (cal. yr BP)**	**GICC05 age (yr b2k)**
**15▲**	*N. pachyderma* s	UBA-21487	2229 ± 27	1836 ± 41	1886 ± 41
**40**	*N. pachyderma* s	UBA-21488	4570 ± 32	4783 ± 53	4833 ± 53
**70**	*N. pachyderma* s	UBA-21489	8083 ± 44	8535 ± 59	8585 ± 59
**130**	*N. pachyderma* s	UBA-21490	10905 ± 50	12452 ± 93	12502 ± 93
**150**	*N. pachyderma* s	UBA-21894	12186 ± 53	13630 ± 117	13680 ± 117
**195**	*N. pachyderma* s	UBA-21595	13493 ± 55	15894 ± 368	15944 ± 368
**230**	*N. pachyderma* s	UBA-21492	15786 ± 79	18628 ± 69	18678 ± 69
**305**	*N. pachyderma* s	UBA-21493	23962 ± 166	28304 ± 202	28354 ± 202
**350**	*N. pachyderma* s	UBA-21494	27459 ± 204	31321 ± 117	31371 ± 117
**430**	*N. pachyderma* s	UBA-21495	33614 ± 412	37971 ± 593	38021 ± 593
**555**	*N. pachyderma* s	UBA-21496	46045 ± 2028	48640 ± 1361	48690 ± 1361

Supplementary Table 4: MS, K/Ti, and δ^{18}O tie-points of Interstadial (IS) onsets in core JM11-FI-19PC and the NGRIP ice core. By re-evaluating the MS and XRF-K/Ti data, the tie-points for IS2, IS7, IS16, and IS17 could be improved compared to their original definition by Ezat *et al*. (ref. 41). The onset of IS1 was improved by also using the δ^{18}O signal of the benthic foraminiferal species *M. barleeanus* and *C. neoteretis* (for data see ref. 41). In addition, the onset of IS8 as identified by Ezat *et al*. (ref. 41) is now also used as tie-point.

Tie points	GICC05 age ± 1σ (yr b2k)	NGRIP depth (m)	Reference (NGRIP)	JM11-FI-19PC depth (cm)	Reference (JM11-FI-19PC)
**Onset IS 1**	14 692 ± 93	1604.64	37, 38	196	This study
**Onset IS 2**	23 340 ± 298	1793.20	38, 42, 48, 49	272	This study
**Onset IS 3**	27 780 ± 416	1869.12	38, 42, 48, 49	313	41
**Onset IS 4**	28 900 ± 449	1891.57	38, 42, 48, 49	323	41
**Onset IS 5**	32 500 ± 566	1951.66	38, 42, 48	348	41
**Onset IS 6**	33 740 ± 606	1974.56	38, 42, 48, 49	362	41
**Onset IS 7**	35 480 ± 661	2009.45	38, 42, 48, 49	390	This study
**Onset IS 8**	38 220 ± 725	2070.03	38, 48, 49	441	41, This study
**Onset IS 10**	41 460 ± 817	2124.03	38, 42, 48, 49	486	41
**Onset IS 11**	43 340 ± 868	2157.49	38, 49	513	41
**Onset IS 12**	46 860 ± 956	2222.30	38, 49	545	41
**Onset IS 13**	49 280 ± 1015	2256.90	38, 49	567	41
**Onset IS 14**	54 220 ± 1150	2345.52	38, 49	590	41
**Onset IS 15**	55 800 ± 1196	2366.32	38, 49	625	41
**Onset IS 16**	58 280 ± 1256	2402.55	38, 49	637	This study
**Onset IS 17**	59 440 ± 1287	2420.44	38, 49	668	This study
**Onset IS 18**	64 100 ± ?	2465.85	37, 49	688	This study
**Onset IS 19**	72 340 ± ?	2535.96	37, 49	716	This study
**Onset IS 20**	76 440 ± ?	2580.13	37, 49	737	This study
**Onset IS 21**	85 060 ± ?	2691.13	49	788	This study

Supplementary References

1. Knutz, P. C., Zahn, R. & Hall, I. R. Centennial-scale variability of the British Ice Sheet: implications for climate forcing and Atlantic meridional overturning circulation during the last deglaciation. *Paleoceanogr.* **22**, PA1207, doi: 10.1029/2006PA001298 (2007).
2. Rasmussen, T. L., Thomsen, E., Troelstra, S. R., Kuijpers, A. & Prins, M. A. Millennial-scale glacial variability versus Holocene stability: changes in planktic and benthic foraminifera faunas and ocean circulation in the North Atlantic during the last 60 000 years. *Mar. Micropal.* **47**, 143–176 (2003).
3. Jonkers, L. *et al.* A reconstruction of sea surface warming in the northern North Atlantic during MIS 3 ice-rafting events. *Quat. Sci. Rev.* **29**, 1791–1800 (2010).
4. Rasmussen, T. L. & Thomsen, E. The role of the North Atlantic Drift in the millennial timescale glacial climate fluctuations. *Palaeogeogr., Palaeoclim., Palaeoecol.* **210**, 101–116 (2004).
5. Rasmussen, T. L., Thomsen, E., Labeyrie, L. & van Weering, T. C. E. Circulation changes in the Faeroe-Shetland Channel correlating with cold events during the last glacial period (58–10 ka). *Geology* **24**, 937–940 (1996).
6. Koç, N., Jansen, E. & Haflidason, H. Paleoceanographic reconstructions of surface ocean conditions in the Greenland, Iceland and Norwegian Seas through the last 14ka based on diatoms. *Quat. Sci. Rev.* **12**, 115–140 (1993).
7. Sarnthein, M. *et al*. Variations in Atlantic surface ocean paleoceanography, 50°–80°N: a time-slice record of the last 30,000 years. *Paleoceanogr.* **10**, 1063–1094 (1995).
8. Stoner, J. S., Channell, J. E. T. & Hillaire-Marcel, C. A 200 ka geomagnetic chronostratigraphy for the Labrador Sea: indirect correlation of the sediment record to SPECMAP. *Earth Planet. Sci. Lett.* **159**, 165–181 (1998).
9. De Vernal, A. & Hillaire-Marcel C. Sea-ice cover, sea-surface salinity and halo-/thermocline structure of the northwest North Atlantic: modern versus full glacial conditions. *Quat. Sci. Rev.* **19**, 65–85 (2000).
10. Hagen, S. & Hald, M. Variation in surface and deep water circulation in the Denmark Strait, North Atlantic, during marine isotope stages 3 and 2. *Paleoceanogr.* **14**, 1061, doi: 10.1029/2001PA000632 (2002).
11. Jessen, S. P. & Rasmussen, T. L. Sortable silt cycles in Svalbard slope sediments 74–0 ka. *J. Quat. Sci*. **30**, 743–753 (2015).
12. Hodell, D. A., Evans, H. F., Channel, J. E. T. & Curtis, J. H. Phase relationships of North Atlantic ice-rafted debris and surface-deep climate proxies during the last glacial period. *Quat. Sci. Rev*. **29**, 3875–3886 (2010).
13. Rasmussen, T. L. & Thomsen, E. Warm Atlantic surface water inflow to the Nordic seas 34–10 calibratesd ka B.P. *Paleoceanogr.* **23**, PA1201, doi: 10.1029/2007PA001453 (2008).
14. Dokken, T. M., Nisancioglu, K. H., Li, C., Battisti, D. S. & Kissel, C. Dansgaard-Oeschger cycles: interactions between ocean and sea ice intrinsic to the Nordic seas. *Paleoceanogr*. **28**, 491–502, doi.10.1002/palo.20042 (2013).
15. Rørvik**,** K. -L., Rasmussen, T. L., Hald, M. & Husum, K. Intermediate water ventilation in the Nordic seas during MIS 2. *Geophys. Res. Lett*. **40**, 1–6, doi:10.1002/grl.50325 (2013).
16. Peck, V. L., Hall, I. R., Zahn, R. & Elderfield, H. Millennial-scale surface and subsurface paleothermometry from the northeast Atlantic, 55–8 ka BP. *Paleoceanogr.* **23**, PA3221, doi: 10.1029/2008PA001631 (2008).

17. Hibbert, F. D., Austin, W. E. N., Leng, M. J. & Gatlif, R. W. British Ice Sheet dynamics inferred from North Atlantic ice-rafted debris records spanning the last 175 000 years. *J. Quat. Sci.* **25**, 461–482 (2010).
18. Hall, I. R., Colmenero-Hidalgo, E., Zahn, R., Peck, V. L. & Hemming, S. R. Centennial-to millennial-scale ice-ocean interactions in the subpolar northeast Atlantic 18–41 kyr ago. *Paleoceanogr.* **26,** PA2224, doi: 10.1029/2010PA002084 (2011).
19. Müller, J. & Stein, R. High-resolution record of late glacial and deglacial sea ice changes in Fram Strait corroborates ice-ocean interactions during abrupt climate shifts. *Earth Plan. Sci. Lett.* **403**, 446–455 (2014).
20. Rasmussen, T. L., Van Weering, T. C. E. & Labeyrie, L. High resolution stratigraphy of the Faeroe-Shetland Channel and its relation to North Atlantic paleoceanography: the last 87 kyr. *Mar. Geol.* **131**, 75–88 (1996b).
21. Cortijo, E. *et al.* Changes in sea surface hydrology associated with Heinrich event 4 in the North Atlantic Ocean between 40° and 60°N. *Earth Plan. Sci. Lett.* **146**, 29–45 (1997).
22. Bond, G. *et al*. Correlations between climate records from North Atlantic sediments and Greenland ice. *Nature* **365**, 143–147 (1993).
23. Fronval, T., Jansen, E., Bloemendal, J. & Johnsen, S. J. Oceanic evidence for coherent fluctuations in Fennoscandian and Laurentide ice sheets on millennium timescales. *Nature* **374**, 443–446 (1995).
24. Barker, S. *et al*. Icebergs not the trigger for North Atlantic cold events. *Nature* **520**, 333–338 (2015).
25. Bauch, H. A. *et al.* A multiproxy reconstruction of the evolution of deep and surface waters in the subarctic Nordic seas over the last 30,000 yr. *Quat. Sci. Rev*. **20**, 659–678 (2001).
26. Stein, R., Nam, S. -I., Grobe, H. & Hubberten, H. Late Quaternary glacial history and short-term ice-rafted debris fluctuations along the East Greenland continental margin. *Geol. Soc. Spec. Publ*. **111**, 135–151 (1996).
27. Nam, S. & Stein, R. in *On the Determination of Sediment Accumulation Rates.* GeoResearch Forum Vol. 5 (eds Bruns, P. & Hass, H.C.) 223–240 (Trans Tech Publications, 1999).
28. Stein, R. *Arctic Ocean Sediments: Processes, Proxies and Paleoenvironment* (Elsevier Science, 2008).
29. Telesinski, M. M., Spielhagen, R. F. & Lind, E. A high-resolution Lateglacial and Holocene palaeoceanographic record from the Greenland Sea. *Boreas* **43**, 273–285 (2015).
30. Voelker, A. H. L. *et al.* Correlation of marine ^{14}C ages from the Nordic seas with the GISP2 isotope record: implications for ^{14}C calibration beyond 25 ka BP. *Radiocarbon* **40**, 517–534 (1998).
31. Müller, J., Massé, G., Stein, R. & Belt, S. T. Variability of sea-ice conditions in the Fram Strait over the past 30,000 years. *Nat. Geosci.* **2**, 772–776 (2009).
32. Rasmussen, T. L., Thomsen, E. & Moros, M. North Atlantic warming during Dansgaard-Oeschger events synchronous with Antarctic warming and out-of-phase with Greenland climate. *Nature Sci. Rep*. **8**, doi:10.1038/srep20535 (2016).
33. Van Kreveld, S. *et al.* Potential links between surging ice sheets, circulation changes, and the Dansgaard-Oeschger cycles in the Irminger Sea, 60–18 kyr. *Paleoceanogr.* **15**, 425–442 (2000).
34. Moros, M. *et al.* Were glacial iceberg surges in the North Atlantic triggered by climate warming? *Mar. Geol.* **192**, 393–417 (2002).

35. Elliot, M. Labeyrie, L. & Duplessy, J. -C. Changes in North Atlantic deep-water formation associated with the Dansgaard-Oeschger temperature oscillations (60–10 ka). *Quat. Sci. Rev.* **21**, 1153–1165 (2002).
36. Oppo, D. W. & Lehman, S. J. Suborbital timescale variability of North Atlantic Deep Water during the past 200,000 years. *Paleoceanography* **10**, 901–910 (1995).
37. Rasmussen, S. O. *et al.* A new Greenland ice core chronology for the last glacial termination. *J. Geophys. Res.* **111**, D06102, doi: 06110.01029/02005JD006079 (2006).
38. Svensson, A. *et al.* A 60 000 year Greenland stratigraphic ice core chronology. *Clim. Past* **4**, 47–57, doi: 10.5197/cp-4-47-2008 (2008).
39. Griggs, A. J. *et al.* Optimising the use of marine tephrochronology in the North Atlantic: a detailed investigation of the Faroe Marine Ash Zones II, III, and IV. *Quat. Sci. Rev.* **106**, 122–139 (2014).
40. Wastegård, S. & Rasmussen, T. L. Faroe Marine Ash Zone IV – a new MIS3 ash zone on the Faroe Islands margin. *Geol. Soc. Spec. Publ*. **398**, 81–93 (2014).
41. Ezat, M. M., Rasmussen, T. L. & Groeneveld, J. Persistent intermediate water warming during cold stadials in the southeastern Nordic seas during the past 65 k.y. *Geology* **42**, 663–666 (2014).
42. Svensson, A. *et al.* The Greenland Ice Core Chronology 2005, 15–42 ka. Part 2: comparison to other records. *Quat. Sci. Rev.* **25**, 3258–3267 (2006).
43. Wastegård, S. & Rasmussen, T. L. New tephra horizons from Oxygen Isotope Stage 5 in the North Atlantic: correlation potential for terrestrial, marine and ice-core archives. *Quat. Sci. Rev.* **20**, 1587–1593 (2001).
44. Abbott, P. M. *et al.* Re-evaluation and extension of the Marine Isotope Stage 5 tephrostratigraphy of the Faroe Islands region: The cryptotephra record. *Palaeogeogr., Palaeoclim., Palaeoecol*. **409**, 153–168 (2014).
45. Davies, S. M. *et al.* A North Atlantic tephrostratigraphical framework for 130–60 ka b2k: new tephra discoveries, marine-based correlations, and future challenges. *Quat. Sci. Rev.* **106**, 101–121 (2014).
46. Stuiver, M. & Reimer, P. J. Extended ^{14}C database and revised CALIB radiocarbon calibration program. *Radiocarbon* **35**, 215–230 (1993).
47. Reimer, P. J. *et al*. IntCal13 and Marine13 radiocarbon age calibration curves, 0–50,000 years cal BP. *Radiocarbon* **55**, 1869–1887 (2013).
48. Andersen, K. K. *et al.* The Greenland Ice Core Chronology 2005, 15–42 ka. Part 1: constructing the time scale. *Quat. Sci. Rev.* **25**, 3246–3257 (2006).
49. Rasmussen, S. O. *et al.* A stratigraphic framework for abrupt climatic changes during the last glacial period based on three synchronized Greenland ice-core records: refining and extending the INTIMATE event stratigraphy. *Quat. Sci. Rev.* **106**, 14–28 (2014).
